# A Newly Identified Frontal Path from Fornix in Septum Pellucidum with 7.0T MRI Track Density Imaging (TDI) – The Septum Pellucidum Tract (SPT)

**DOI:** 10.3389/fnana.2015.00151

**Published:** 2015-11-27

**Authors:** Zang-Hee Cho, Je-Geun Chi, Sang-Han Choi, Se-Hong Oh, Sung-Yeon Park, Sun Ha Paek, Chan-Woong Park, Fernando Calamante, Young-Bo Kim

**Affiliations:** ^1^Neuroscience Research Institute, Gachon University of Medicine and ScienceIncheon, South Korea; ^2^Department of Pathology, Seoul National University College of MedicineSeoul, South Korea; ^3^Department of Radiology, School of Medicine, University of Pennsylvania, PhiladelphiaPA, USA; ^4^Departments of Neurosurgery, Seoul National University College of MedicineSeoul, South Korea; ^5^The Florey Institute of Neuroscience and Mental Health, MelbourneVIC, Australia; ^6^Department of Medicine, Austin Health and Northern Health, University of Melbourne, MelbourneVIC, Australia

**Keywords:** 7.0T MRI, track density imaging, septum pellucidum tract, fornix, fiber tracking, tractography, diffusion MRI

## Abstract

The high anatomical contrast achieved with the newly emerging MRI tractographic technique of super-resolution track density imaging (TDI) encouraged us to search for a new fiber tract in the septum pellucidum. Although this septum pellucidum tract (SPT) has been observed previously, its connections were unclear due to ambiguity and limited resolution of conventional MRI images. It is now possible to identify detailed parts of SPT with the increased resolution of TDI, which involves diffusion MRI imaging, whole-brain tractography, and voxel subdivision using the track-count information. Four healthy male subjects were included in the study. The experiment was performed with 7.0T MRI, following the guidelines of the institute’s institutional review board. Data were processed with the super-resolution TDI technique to generate a tractographic map with 0.18 mm isotropic resolution. The SPT was identified in all subjects. Based on additional seed tracking method with inter-axis correlation search, we have succeeded in identifying a new frontal lobe pathway in the SPT. We hypothesize that the tract is connected as a superior dorsal branch of the fornix that leads to the prefrontal cortex.

## Introduction

Despite substantial progress with diffusion tensor imaging (DTI; Basser et al., 1994) in MRI in the last two decades, its sensitivity to accurately image white matter tracts in the brain remains somewhat limited (Tournier et al., 2011). More recently, however, several new post processing imaging techniques have been introduced (Tournier et al., 2007; Calamante et al., 2010, 2011), including super-resolution track density imaging (TDI), which was shown to greatly increase the sensitivity for visualization of white matter structures (Calamante et al., 2010). Sensitivity is increased even further when these methods are combined with ultra-high field MRI (Calamante et al., 2012, 2013; Kurniawan et al., 2014; Richards et al., 2014; Cho et al., 2015a,b; Ullmann et al., 2015). These new methods therefore encouraged us to search for a fiber tract in the septum pellucidum, which is difficult to visualize *in vivo* due to the limited resolution, contrast and sensitivity of current DTI MRI images.

**FIGURE 1:**
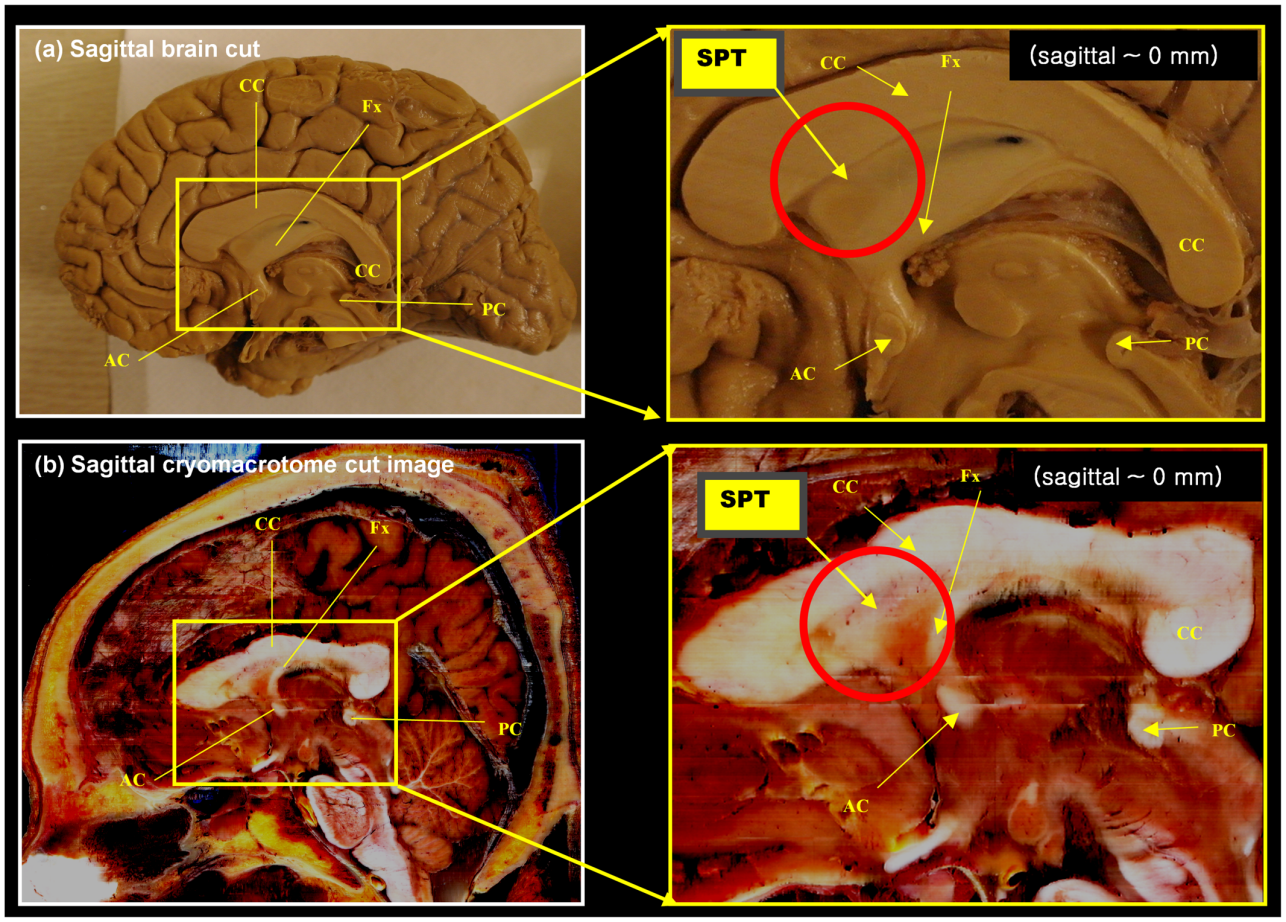
**Anatomy of the septum pellucidum area (red circle). (a)** Mid-sagittal brain image (sagittal, 0 mm) with its extended region of interest (ROI) image (yellow box). **(b)** Middle sagittal cryomacrotome image with its extended ROI image (yellow box). Although the septum pellucidum tract (SPT) has been unknown until now, it does subtly appear in these images: the SPT can be seen as shown in **(a)**. It is also visible in **(b)** as a dorsal branch of the fornix in the frontal area of the septum pellucidum. AC, anterior commissure, CC, corpus callosum, Fx, fornix, PC, posterior commissure.

**FIGURE 2:**
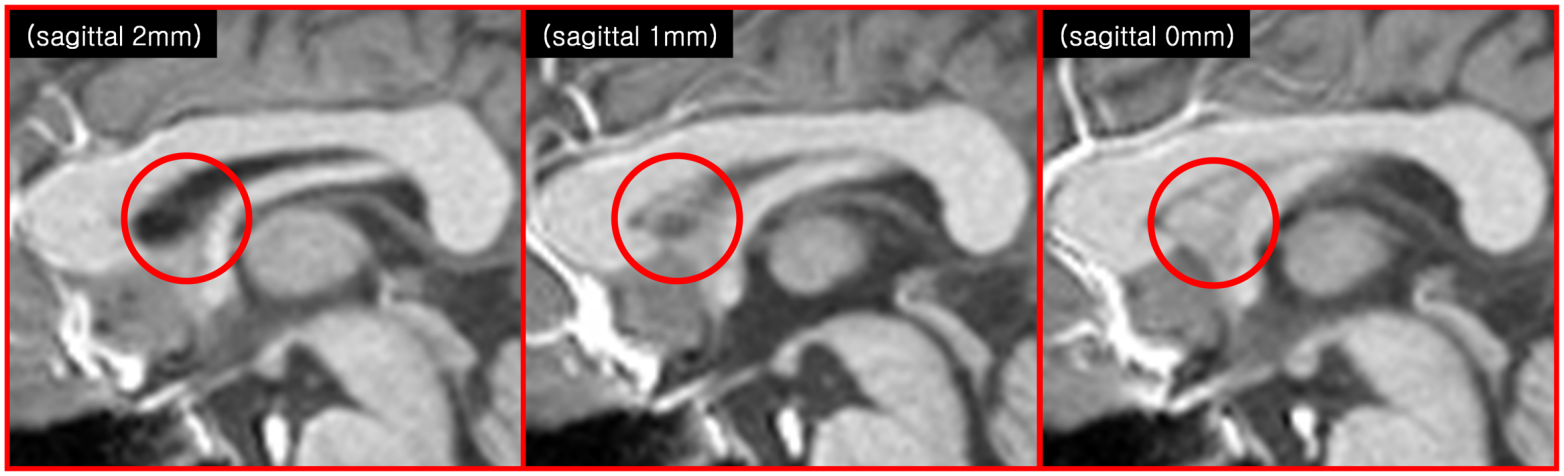
**Another indication of the SPT is seen in the anatomical images of modern MRI scanners (3T T1-weighted sagittal MRI images).** The images at 1 and 0 mm show some faint structures in the septum pellucidum area (red circle), but not in the image at 2 mm.

A set of anatomical images of brain structures with the fornix in the septum pellucidum is shown in **Figures 1 and 2**. These anatomical images are interesting because although some tissues or fibers seem visible in the septum pellucidum, no detail description has been paid to these fibers (see for example figure on page 24 of the Brain Atlas, by Hanaway et al., 1990). Similarly, another interesting clue toward this structure is given by the DTI atlas of Mori et al. (2005) and Oishi et al. (2010), in which they showed some indications that there may be fibers extending from the crus of the fornix to the anterior and dorsal aspect of the septum pellucidum. While some faint images of dorsal branches of the fornix are visible in these images, they are difficult to recognize, and there is in fact no mention of the existence of such a structure. Recently, there was a paper describing this fiber in the septum pellucidum (Cho et al., 2015b), but the connections from this fiber were still unclear. Based on data from additional subjects, and further analysis using a seed-based tracking method, in this study we have identified and confirmed additional connections from this fiber (the septum pellucidum tract, SPT) to frontal areas. It is hypothesized that this new structure could be a potential new target for neurosurgery, such as for deep brain stimulation (DBS) where many emerging targets are being sought.

## Materials and Methods

**FIGURE 3:**
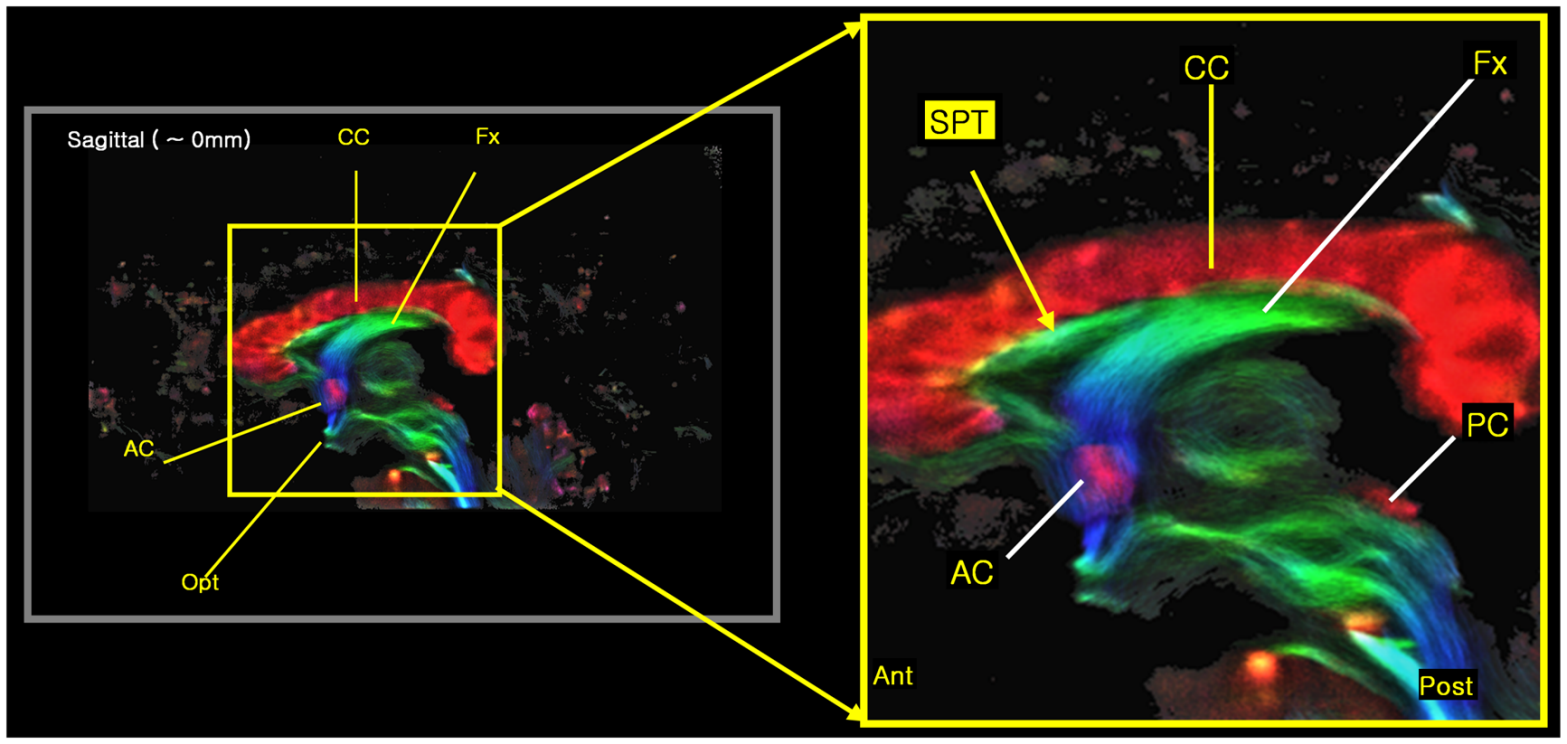
**The new 7.0T super-resolution DEC TDI began to clearly show the tract at the septum pellucidum area in a sagittal view image, especially with its expanded image at right side.** The color-coding indicates the main local orientation (red: left–right, green: anterior–posterior, blue: inferior–superior).

The new STP fiber was identified by super-resolution TDI maps obtained from ultra-high field diffusion MRI data (e.g., see **Figure 3**), and was further confirmed by the seed-based tracking approach and an inter-axis correlation search (Cho et al., 2015b). The inter-axis correlation refers to a manual method for co-localizing structures seen on TDI maps using perpendicular lines placed on coronal, sagittal, and axial images; intersecting inter-axis lines are then manually and visually correlated between the orthogonal planes. Data acquisition and processing therefore consisted of four steps: diffusion weighted imaging (DWI) data acquisition, TDI data processing, seed-based tracking, and the inter-axis correlation search.

Diffusion weighted imaging data were obtained on a 7.0T MRI scanner (Magnetom 7.0T, Siemens, Erlangen, Germany), and the experiment was performed following the guidelines and with the approval of the institute’s institutional review board and Korea food and drug administration. Four young healthy male subjects (age: 24–30 years) were recruited as volunteers, and no one had a record of any health related problems. DWI data were acquired using a single-shot diffusion-weighted echo planar imaging (EPI) sequence with the following parameters: TR/TE = 6,000/83 ms, matrix size = 128 × 128, field-of-view 230 mm × 230 mm, 45 slices, 1.8 mm isotropic resolution, 64 diffusion-weighted directions, *b*-value = 2,000 s/mm^2^, one *b* = 0 image, GRAPPA with factor 3, bandwidth 1562 Hz/pixel, and three repeats, for a total acquisition time of 19 min 0.5 s.

Diffusion weighted imaging data were then processed to calculate super-resolution TDI maps, which involves constrained spherical deconvolution (Tournier et al., 2007) to calculate the fiber orientation distribution (FOD) in each voxel, whole brain probabilistic fiber-tracking, and track-count mapping with voxel sub-division (Calamante et al., 2010). The TDI analysis was carried out using the MRtrix software program (Brain Research Institute, Florey Neuroscience Institutes, Melbourne, Victoria, Australia, http://www.brain.org.au/software/mrtrix/index.html) (Tournier et al., 2012). Relevant tracking parameters were: tracking type = SD_PROB, track minimum length = 20 mm, step-size = 0.02 mm, curvature radius constraint = 0.04 mm, FOD cutoff for track termination = 0.3, and number of tracks = 6,000,000 (Tournier et al., 2012). The final TDI image was generated with a nominal isotropic resolution of 0.18 mm, i.e., corresponding to a voxel size 1/1000 smaller than the acquired DWI data. These TDI maps were color-coded according to the direction of the fibers (based on the mean color of all streamline segments within the voxel (Calamante et al., 2010), i.e., with red: left-right, green: anterior-posterior, and blue: inferior-superior), to generate the so-called directionally-encoded color (DEC) TDI maps.

**FIGURE 4:**
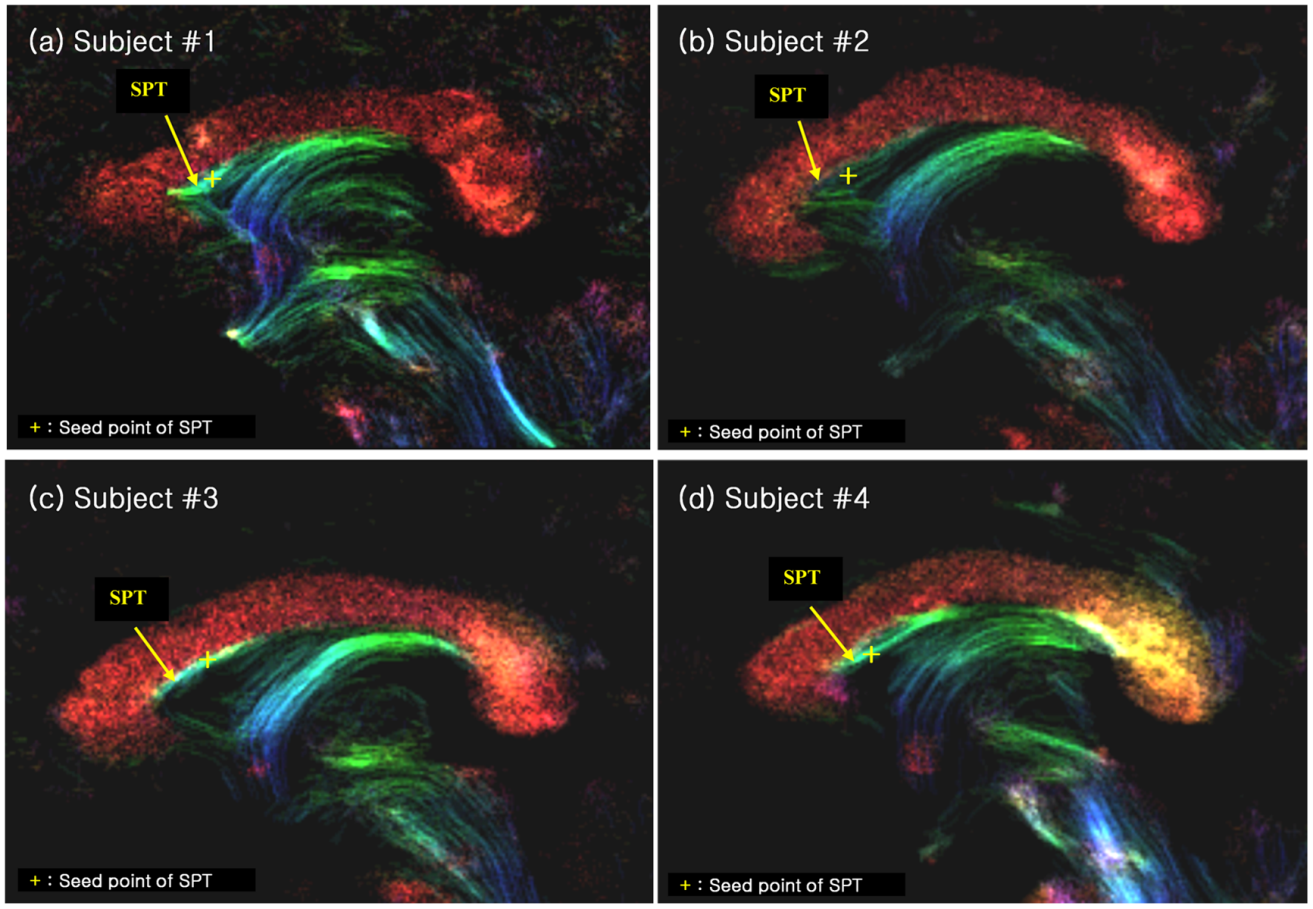
**The SPT on the super-resolution DEC TDI of four different subjects (labelled as **(a)**, **(b)**, **(c)** and **(d)**, respectively).** The SPTs are indicated by yellow arrows and yellow crosses. The latter are used as the seed points for the search of the SPTs and its connections (see **Figure 5**). The color-coding indicates the main local orientation (red: left–right, green: anterior–posterior, blue: inferior–superior).

We have also performed seed-based tracking analysis to further identify and characterize the SPT, particularly to identify the connection to forebrain areas. We selected a single voxel seed point or region of interest in the SPT, which lied in the medial part of sagittal view image (sagittal slice between -1 mm and +1 mm), as shown with yellow crosses in **Figure 4** (see Results section). Based on the SPT seed, the ‘streamtrack’ function in MRtrix was used for probabilistic seed-based tracking, with the following relevant parameters: tracking type (SD_PROB), track minimum length = 20 mm, step-size = 0.02 mm, curvature radius constraint = 0.08 mm, number of tracks = 1,000 (Tournier et al., 2012).

To further confirm the seed-based tracking results and to help with the identification of the SPT fiber, we performed an additional inter-axis correlation search (Cho et al., 2015b) in the sagittal and axial views.

## Results

We found several interesting features of the SPT based on the current study. First, the new fiber, which was observed clearly in all four subjects, appeared to be a dorsal branch of the fornix (**Figure 4**). SPT branches were clearly visible and differentiated from the main body of the fornix as well as from the corpus callosum (e.g., see expanded image in **Figure 3**, where the SPT can be seen as the green structure adjacent to the corpus callosum in a mid-brain sagittal slice); the distinction between the SPT and surrounding structures was greatly facilitated by the high resolution and high contrast of the DEC-TDI maps. In particular, in the sagittal view, the SPT appeared to originate from the caudal aspect or crus of the fornix and extend toward the genu of the corpus callosum, as if it were an anterior dorsal branch of the fornix at the septum pellucidum. The SPT is implicitly shown in **Figures 1** and **2**; however, no specific reference has ever been mentioned regarding this fiber (Hanaway et al., 1990; Mori et al., 2005; Oishi et al., 2010). Moreover, specific mention of the presence of additional fiber distribution in this area would have been extremely difficult to make solely based on anatomical images such as those shown in **Figures 1** and **2**, due to limited contrast and/or resolution.

**FIGURE 5:**
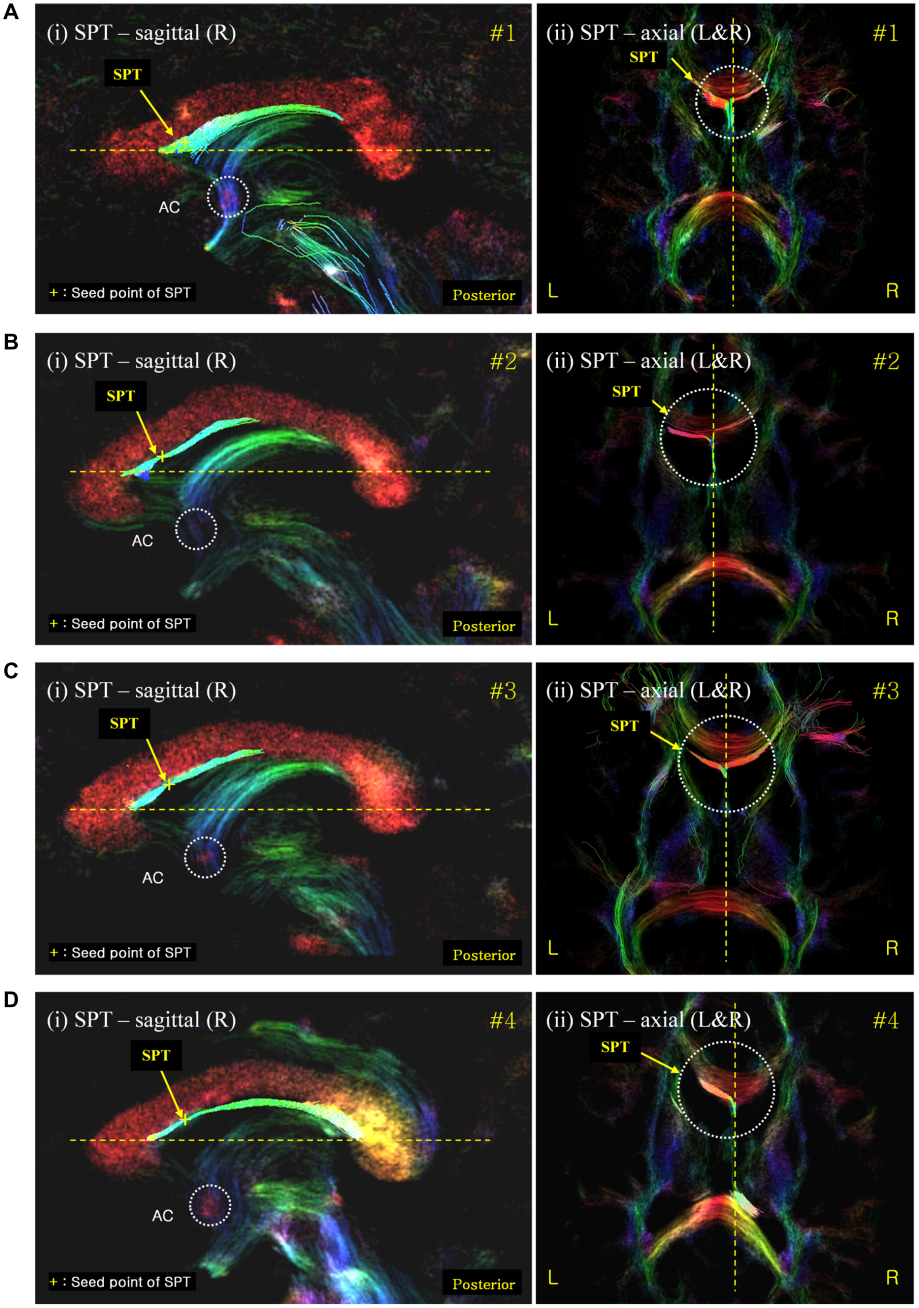
**Septum pellucidum tract seed-based tracking results shown with correlated sagittal and axial view images. (A)** The SPT seed-based tracking result from subject #1; **(B–D)** equivalent results from subjects #2, #3, and #4, respectively. The left [also labeled (i)] and right (ii) columns show the seed-based tracking results in sagittal view images and their corresponding axial view images, respectively. The tracks displayed correspond to those within a 0.54 mm slab (i.e., also including the tracks in the surrounding slices) overlaid on the DEC-TDI map. The yellow cross on the SPT of the sagittal view images (left) indicates the seed point positions in each data-set. The color-coding of the background image indicates the main local orientation (red: left–right, green: anterior–posterior, blue: inferior–superior). AC, anterior commissure (as indicated by the dashed circle in the sagittal images). The dashed circle in the axial images highlights the area of the SPT at the anterior end of the septum pellucidum where it splits laterally.

Second, the SPT seems to be directed toward the prefrontal area from the frontal end of the septum pellucidum (**Figures 5A–D**). In the mid-sagittal view [**Figure 5A**(i)] in subject #1, as well as for the other subjects in **Figures 5A–D**, the SPT abruptly reaches the anterior end of the septum pellucidum or immediately behind the genu of the corpus callosum, splits laterally, and it is eventually directed toward prefrontal areas. SPT presents only in the midline of the septum pellucidum and more or less disappears as it leaves the midline or the septum pellucidum (only within -3 mm to +3 mm from the midline). We were able to trace the forwardly directed SPT tract as shown in **Figure 5** in the axial view images, and found that the SPT was split laterally at the genu or end of the anterior aspect of the SPT, and appears directed toward the prefrontal area, particularly toward the superior frontal area, by following the genu of the corpus callosum (Note: these projections toward the superior frontal area cannot be visualized in the specific planes shown in **Figure 5**). The SPT seems to circle around the corpus callosum and merge with the anterior limb of the internal capsule or minor forceps.

**FIGURE 6:**
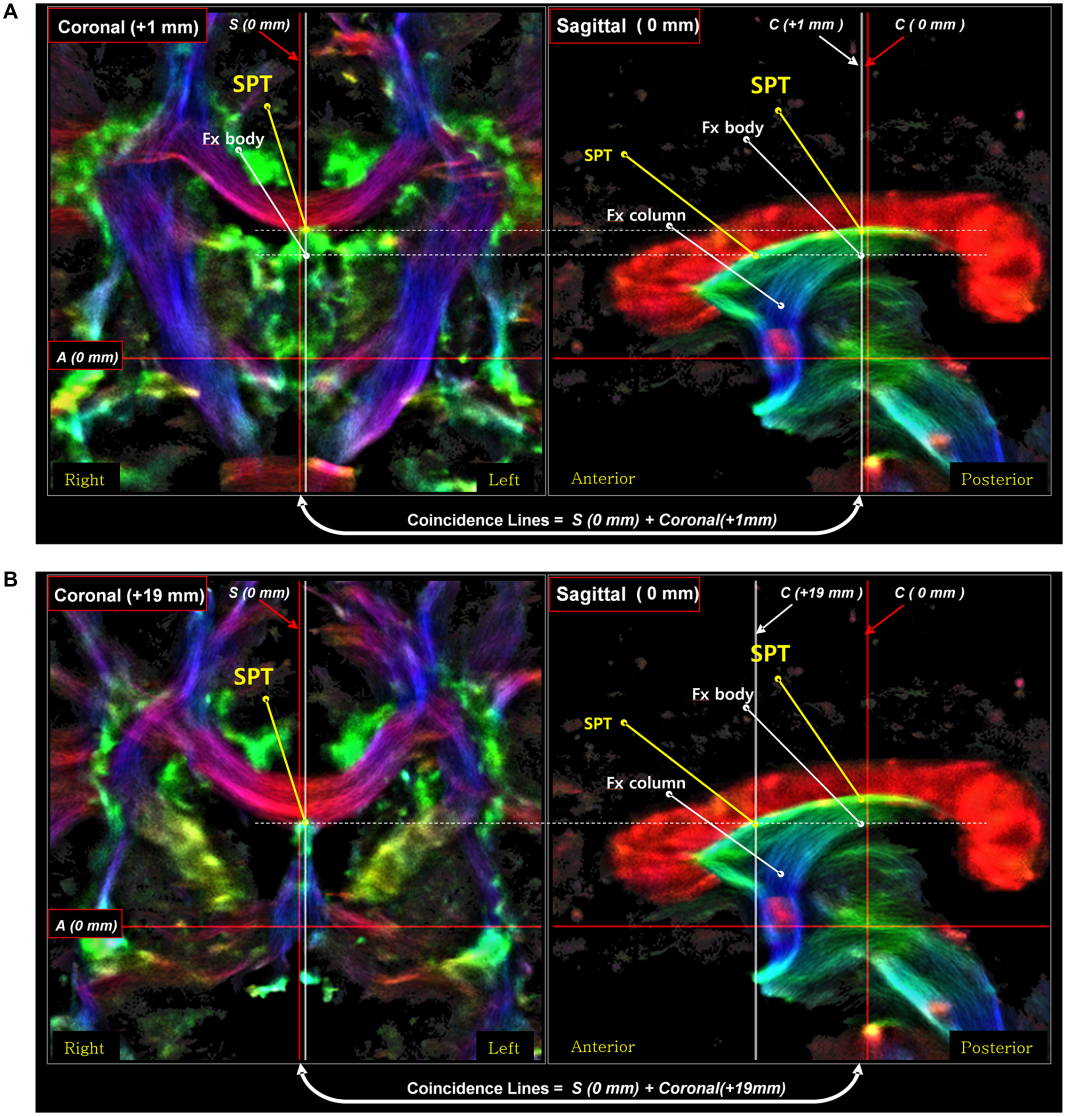
**An inter-axis correlation search for the corroboration of the SPT in a sagittal view image with two coronal view images on the left [coronal image at 1 mm **(A)** and coronal image at 19 mm **(B)**].** A set of coronal-sagittal super-resolution DEC TDI maps was selected, and an inter-axis correlation study was conducted. The SPT is clearly visible both in the coronal and sagittal views. The thick white lines are the coincidence lines where the values are the same in both images. The result of this correlation search suggests that the SPT is a new separate dorsal-anterior branch of the fornix (Fx), different from the main body of the fornix, the precommissural fornix. Part of this figure has been modified from a figure previously published in Cho et al. (2015b), with permission from Elsevier.

Third, the posterior part of the SPT is connected to the body or crus of fornix (**Figure 6**). To confirm the connection of the SPT to the main body of the fornix, we examined the area using an inter-axis correlation search (Cho et al., 2015b) with coronal view data at 1 mm (**Figure 6A**) and 19 mm (**Figure 6B**), respectively, with the midline sagittal view data at 0 mm. The SPT is separated from the superior dorsal branch of the fornix at the caudal aspect (**Figure 6A**) and the SPT distinguishes itself from the other part of the fornix in the anterior aspect, such as the pre-and post-commissural fornix as shown in **Figure 6B**.

## Discussion

Although there have been a large number of studies about the septum pellucidum, the fiber in the septum pellucidum has not been well described so far, and the connection from this area is at best unknown based on anatomical and tractography approaches. The septum pellucidum is a thin vertical sheet of nervous tissue that is covered on either side by the ependyma. The ependyma forms a single layer of cuboidal or columnar cells that are different from the fiber (Richard, 2001). However, in some reports, the medial surface of the septum pellucidum is claimed to be some form of white matter structure (Swayam et al., 2006; Marinković et al., 2009). In this study, guided by the super-resolution TDI technique from ultra-high field 7T MRI, we have clearly observed the existence of the fiber in the septum pellucidum and identified its frontal connection, which were further confirmed using an inter-axis correlation search between coronal and sagittal view image data.

The fornix is one of the major fibers in the limbic system with numerous functional connections; however, no direct connections to prefrontal areas have been found other than the septal nucleus and the nucleus accumbens. Based on previous studies, the pre-commissural fornix is well known, both from human histological studies as well as *in vivo* MRI/DTI studies; however, little is known about the SPT and even its connections. This newly identified SPT appears as a dorsal branch of the fornix and connected to the prefrontal area. The SPT is not only connected to the fornix but also to the prefrontal cortex, as shown in **Figure 5**, where the two bifurcated branches of the SPT are connected toward the prefrontal cortex. We hypothesized that the SPT could be an important and unique route for the direct connection of the hippocampus or hippocampal complex, which consists of the para-hippocampal gyrus to entorhinal cortex, to the prefrontal cortex, which includes the dorsolateral prefrontal cortex and septal nuclei, through the fornix; a connection that has not been observed previously. Future studies are required to validate and further characterize this possible new connection. Until those studies are carried out, the exact extent of the apparent frontal connections identified in the current study should be considered as preliminary findings.

Importantly, considering the fact that the SPT is connected to the septal nuclei and possibly to the dorsolateral prefrontal cortex, it could play an important role in controlling the cognitive and attractive activity such as the sorrow and pleasure. We hypothesize that this structure could therefore be a new target for external intervention, such as by DBS, which has begun to be applied in treatment of major depressive disorders or other cognitive diseases, such as the Alzheimer’s and obsessive and compulsive disorder, or even post-traumatic stress disorder (PTSD; Srejic et al., 2015). Future studies are required to test this hypothesis.

Interestingly, the SPT defined here was highlighted as a structure just above the fornix with lower fractional anisotropy (FA) in patients with Alzheimer’s disease compared with cognitively normal individuals in a voxel-based analysis (see sagittal view of Figure 2A in Oishi et al., 2011). This provides further evidence of the potential important clinical significance of this newly identified structure.

### Technical Issues

While ultra-high field MRI was used in the current study, other suitable studies at lower field strengths, such as 3T, should be also possible to visualize in principle the SPT given it is a relatively large structure; identification and visualization is facilitated now based on the results presented here, which have demonstrated that the structure exists and where it is located. For example, similar super-resolution DEC-TDI maps at 3T, or high-resolution DTI maps based on multi-shot acquisitions (Porter and Heidemann, 2009) should be suitable to identify the SPT at 3T. It should be noted, however, that the latter acquisition approach is typically associated with much longer acquisition times, and therefore might be less suited for clinical applications; this is in fact a benefit of the TDI method, where the extra time is spent on post-processing, without necessarily extending the acquisition time.

The choice of super-resolution level is a compromise between several factors, including processing time (the higher the resolution the more tracks are required), output file size and memory requirements (the DEC-TDI data sets shown here were >1 GB in size), and the size of the structure of interest (smaller structures require higher super-resolution). The 0.18 mm voxel dimension (corresponding to a 1/10 super-resolution in each orientation) was found to be a suitable compromise. As mentioned above, given the SPT is a relatively large structure, lower super-resolution levels are likely to have been sufficient for identification; however, as shown in Figure 1 of Calamante et al. (2010), the structure’s conspicuity and sharpness would have been lower.

Finally, it should be noted that diffusion MRI fiber-tracking does not directly visualize the axonal fibers but rather infers an estimate of the location and orientation of white matter bundles based on the diffusion of water molecules. Diffusion MRI fiber-tracking is subject to a number of well-known limitations and possible sources of error, including the effect of ‘kissing’ and ‘crossing fibers,’ the effect of noise, partial volume, etc. (Tournier et al., 2011). To minimize these sources of error, we used here state-of-the art methods, including advanced models to characterize multiple fiber populations and a probabilistic fiber-tracking algorithm (see Materials and Methods section). It is important to emphasize, however, that validation of fiber-tracking methods remains an area of major challenge and great interest in the field of diffusion MRI, and results from fiber-tracking based methods should be considered with caution since they can be influenced by many technical issues.

## Author Contributions

1. Substantial contributions to the conception or design of the work (Z-HC, J-GC, S-HP, FC, Y-BK).
2. Substantial contributions to the acquisition of data (S-HC, S-HO, S-YP).
3. Substantial contributions to the analysis of data (Z-HC, J-GC, C-WP, FC).
4. Substantial contributions to the interpretation of data (Z-HC, J-GC, S-HP, C-WP, FC).
5. Drafting the work or revising it critically for important intellectual content (all authors: Z-HC, J-GC, S-HC, S-HO, S-YP, S-HP, C-WP, FC, Y-BK).
6. Final approval of the version to be published (all authors: Z-HC, J-GC, S-HC, S-HO, S-YP, S-HP, C-WP, FC, Y-BK).
7. Agreement to be accountable for all aspects of the work in ensuring that questions related to the accuracy or integrity of any part of the work are appropriately investigated and resolved (all authors: Z-HC, J-GC, S-HC, S-HO, S-YP, S-HP, C-WP, FC, Y-BK).

## Conflict of Interest Statement

FC is co-inventor in a patent application on the TDI method. The authors declare that the research was conducted in the absence of any commercial or financial relationships that could be construed as a potential conflict of interest.
